# On enabling collaborative non-intrusive load monitoring for sustainable smart cities

**DOI:** 10.1038/s41598-023-33131-0

**Published:** 2023-04-21

**Authors:** Yunchuan Shi, Wei Li, Xiaomin Chang, Ting Yang, Yaojie Sun, Albert Y. Zomaya

**Affiliations:** 1 School of Computer Science, The University of Sydney, Camperdown, 2006 Australia; 2 School of Electrical and Information Engineering, Tianjin University, Tianjin, 300072 China; 3 School of information science and technology, Fudan University, Shanghai, 200433 China

**Keywords:** Computer science, Computational science, Electrical and electronic engineering, Sustainability

## Abstract

Improving energy efficiency is a crucial aspect of building a sustainable smart city and, more broadly, relevant for improving environmental, economic, and social well-being. Non-intrusive load monitoring (NILM) is a computing technique that estimates energy consumption in real-time and helps raise energy awareness among users to facilitate energy management. Most NILM solutions are still a single machine approach and do not fit well in smart cities. This work proposes a model-agnostic hybrid federated learning framework to collaboratively train NILM models for city-wide energy-saving applications. The framework supports both centralised and decentralised training modes to provide a cluster-based, customisable and optimal learning solution for users. The proposed framework is evaluated on a real-world energy disaggregation dataset. The results show that all NILM models trained in our proposed framework outperform the locally trained ones in accuracy. The results also suggest that the NILM models trained in our framework are resistant to privacy leakage.

## Introduction

Approximately 55% of the world’s population lives in urban areas, and the percentage is expected to increase to 68% by 2050^1^. With the continued expansion of cities, it has become increasingly crucial to manage available resources to cater to the sustainability of urban systems for meeting the ever-increasing needs of the urban population. The recent advancements in the Internet of Things, edge computing, and machine learning provide hardware and software support for paving the way toward sustainable smart cities^2^. One of the grand challenges of realising sustainable smart cities is to address the increasing demand for electrical energy. Various approaches^3–5^ have been developed to overcome this difficulty, but the common element of these approaches is to let consumers be aware of their detailed electricity consumption. Previous studies^6,7^ show that appliance-level information can help reduce energy consumption by raising consumer awareness and facilitating new energy-saving applications for sustainable smart cities.

The energy consumption of individual appliances can be obtained by using Non-Intrusive Load Monitoring (NILM), a computational method to identify appliance status and extract appliance-level electricity consumption from aggregated power data. The aggregated data is only monitored at a single central point, such as the electricity meter of a building or a house. NILM can provide the fine-grained energy consumption information needed by smart grid systems, an essential part of smart cities, to form a cohort for better service delivery. It provides online feedback on the energy consumption of households to let users be well aware of the situations and help them to change use patterns when needed. This information can also help to develop demand response strategies on the grid side for optimising power generation and dispatching. These pairwise interactions promote the progress of smart cities, energy saving, and sustainable development. Over the years, various experimentally feasible solutions have been developed using hidden Markov models, temporal motif mining, or other combinatorial optimisation techniques. Researchers have recently turned their attention to machine learning models due to their superior performance in various applications across multiple disciplines. Many deep learning-based algorithms^8–10^ and gradient boosting algorithms^11,12^ have been developed for NILM applications and outperformed the traditional models in terms of accuracy and efficiency.

Most existing NILM approaches still face significant challenges, hindering their widespread use for sustainable smart cities. First, NILM models need considerable training data to learn representative statistical characteristics to gain high performance. Conventional approaches address this problem by collecting data from stakeholders for centralised model training, with potentially costly data transfers and privacy and security issues precluding them from practical use. In recent years, federated learning was proposed^13^ to train a global model collaboratively without exchanging the raw data of stakeholders. The existing NILM federated learning solutions are deep learning oriented in a centralised setting^14–16^. The central server coordinates all the stakeholders to train a neural network model. These methods can achieve desired performance in experiments but are error-prone in real-world scenarios. Centralised federated learning generally experiences poor scalability due to the resource constraints turning the central node into a performance bottleneck when handling large clients. The complex structure of the deep learning model and the associated hyperparameters also impose a high computational overhead in training and inference, making it less suitable for running on resource-limited devices. In addition, the client data distribution is generally assumed to be non-independent and identical distribution (non-IID) since it is highly inconsistent in quantity and distribution. The non-IID distribution can potentially contribute different update factors to the client models and leads to poor global model fitting^17^. Recent works have attempted to address these issues through transfer learning and filter pruning^18^. These works cannot fundamentally change the nature of deep learning models that require extensive data and computing power for the training. Second, most studies^10,19,20^ focus on long-term (more than one hour) energy disaggregation, which naturally requires a long sequence of main readings for each analysis. The analytic devices need substantial storage space to manage such long readings. Lastly, the data for training NILM models is the electrical consumption readings collected from users and sampled in near real-time. The readings contain the instrumental activities of all appliances, including on and off and operating mode switching. Previous works^21–23^ show that using an off-the-shelf statistical approach, it is technically possible to disclose users’ usage patterns and behaviours from the readings, such as sleeping routines, dinning routines, etc. The current approaches rely heavily on encryption and differential privacy techniques to prevent data leakage^24,25^. The inevitable extra computational cost in model training is introduced to the system and even degrades the model performance at runtime. In addition, a city includes users with different behaviours and activities. The data from these users may have different statistical distributions. There is no simple, cost-effective and secure way of putting all these data together and letting them work as a whole.

In this work, we propose a model-agnostic hybrid federated learning framework for NILM applications to address the above challenges in sustainable smart cities. By hybrid, we mean that our framework supports both centralised and decentralised federated learning modes. The major difference between them is to use a server to coordinate the model training in the centralised mode, while no such server is involved in the decentralised mode. In the decentralised mode, clients are connected through a decentralised network. Each client performs local model training and aggregates models from other clients. An asynchronous model aggregation mechanism can also be employed to refine the training protocol on the fly, providing further flexibility to the system. Under the dual support of training modes, our framework can offer the desired environment to end-users for acquiring performance, scalability, robustness or a combination of them for their NILM applications. By model-agnostic, we mean our framework supports the training of neural network models and gradient boosting decision tree (GBDT) models. Neural network models achieve state-of-the-art performance in energy decomposition, and their training process fits well in distributed learning. These models generally require considerable computing resources for training such scenarios. Some recent works also showed that they could experience privacy leakages during the run, making them not the one-for-all solution to support NILM applications. GBDT, on the other hand, inherits the simple structure of tree models and share fewer parameters during training thus becoming more resource-friendly and secure. The use of GBDT in our framework is motivated by its prior results in non-linear regression problems with low computation complexity^26,27^. Our framework considers the non-independent and identical distributions (non-IID) data between clients on model performance by clustering users with similar energy consumption distribution into one training cluster. We also introduce a short-term energy disaggregation strategy to our framework by shrinking the window size used in the sequence-to-point analysis. This strategy can significantly reduce data management costs at local devices while making real-time decision making on energy management possible.

The main contributions of this paper include:

1. We propose a model-agnostic hybrid federated learning framework to provide a flexible, efficient and secure means to train NILM models in sustainable smart cities. It supports the training of deep neural networks and gradient boosting tree models in the centralised federated learning mode and deep neural networks in the decentralised federated learning.
2. The performance of the proposed framework is empirically evaluated on a real world energy dataset. The results show that NILM models trained in our proposed framework for all training modes outperform those locally trained models in terms of accuracy.
3. We conduct extensive experiments to study the effectiveness of a state-of-the-art gradient attack method against our federated learning framework with NILM applications. We find that our proposed framework can protect user privacy from gradient attacks with promising results.

## Methods

In this section, we present the design of our proposed hybrid federated learning framework for NILM applications.

**Figure 1:**
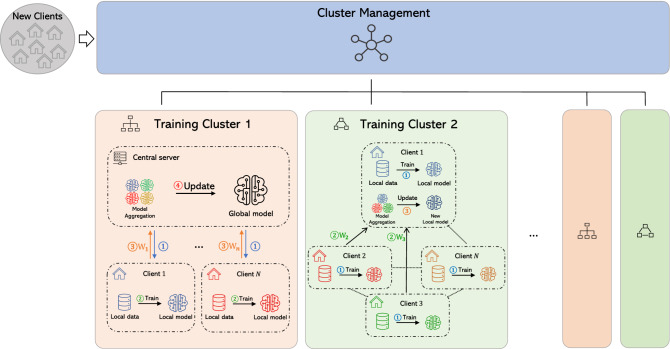
The design of our proposed hybrid federated learning framework for NILM applications.

### An overview of the proposed framework

We aim to propose a model-agnostic hybrid federated learning framework for city-wide NILM applications. The framework, as shown in Fig. 1, first groups clients into clusters according to their similarity in electricity usage and their computation resources. The appropriate federated learning mode (centralised or distributed) and the best-suited machine learning model are determined for each training cluster. Our framework can now support the training of deep neural networks and gradient boosting tree models in the centralised federated learning mode and deep neural networks in the decentralised federated learning mode. We develop a short-term energy decomposition strategy that analyses low-frequency power reading by reducing the window size used in sequence-to-point. The short-term strategy can support real-time energy management decisions, reduce data management costs, and depend less on hardware capabilities.

### Cluster management

It is impractical to expect the users’ consumption data to be always independent and identically distributed (IID) in federated learning scenarios. The locally computed gradients are likely the biased estimates of global gradients, which poses challenges to faster convergence and better performance. To address such a non-IID challenge, we perform clustering over different clients and group users with similar statistical patterns into the same cluster for model training. Our clustering approach also accounts for privacy-preserving by exchanging the Markov transition probabilities rather than raw load measurements. Inspired by Markov Transition Field (MTF)^28^, we convert the clients’ time-series load measurements into Markov matrices. The input space of power consumption sequence $\{x_1 \ldots x_n\}$ is discretised as Q quantile bins, and each element of the sequence is assigned to a quantile. For example, $q_i$ and $q_j$ ($q\in [1,Q]$) denote the quantiles of $x_i$ and $x_j$. The element $M_{ij}$ of the Markov matrix *M* can be calculated by the transition probability from $q_i$ to the quantile $q_j$. With the Markov matrices from the engaged clients, the clustering phase can then be accomplished using TS-SOM (Tree structured self-organizing maps)^29^. TS-SOM divides the generated matrices into multiple groups as a hierarchical clustering method by mapping each tree node to a standard SOM neural network. The clustering is iteratively performed from the root to the leaves until the pre-set tree depth is reached. At the bottom level of the tree, each leaf represents a group of clients that will collaboratively train a NILM model.
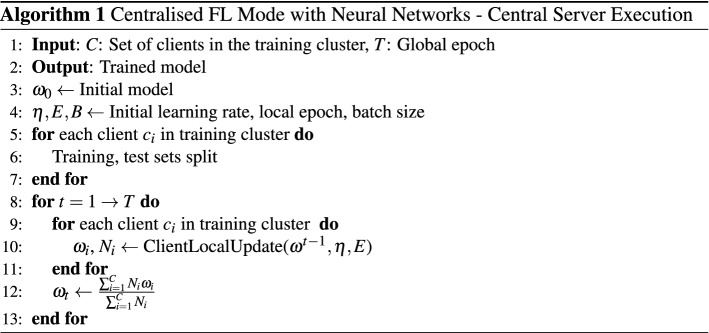

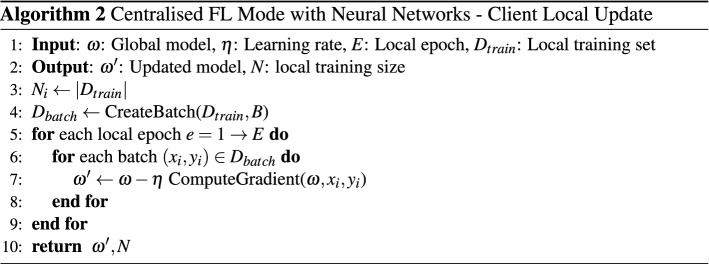


### Centralised federated learning mode

In the centralised federated learning mode, the model training process of each cluster is coordinated by a central server hosted by a trusted third party. Each client maintains a local model for each appliance in this mode and updates the model with its locally available data. Meanwhile, the central server maintains a global model for each appliance and updates the global models by aggregating the updated local models from all corresponding clients. We further introduce the procedures of training deep neural networks and gradient boosting trees in the centralised federated learning mode below.

#### Neural network

Training a deep neural network model in centralised federated learning mode consists of two parts: server execution and client local updates. In client local updates, all clients train the model in parallel and pass the updated model to the server at the end of the training process. The server execution is performed throughout the training process and continuously aggregates the locally updated model. The server execution starts by the central server initialises the global models $\omega _{0}$ while defining the training protocol based on the available computational resources. The training protocol defines the training-test split, learning rate $\eta$, local training batch size *B*, and local training epochs *E*. Each client prepare itself for training by splitting its local data set into a training set and a test set according to the training protocol. The training set is further divided into $\frac{N}{B}$ training batches where *N* is the size of the training set. Within each training iteration *t*, each client performs local update in parallel. Clients first request the latest global model(s) from the central server to update their local model(s). Each client trains the local models using its training data set for *E* epochs. When local training is completed, the performance of the updated local model is then evaluated on the test set. The evaluation result and the updated local model are sent to the central server for updating the global model. The central server updates the global model $\omega _{t}$ through the federated averaging algorithm that performs a weighted aggregation of all clients’ models. Each model is assigned a weight $\frac{N_{i}}{ \sum _{j}^{C}N_{j}}$, where $n_i$ denotes the number of data owned by client *i*, *C* is the number of clients in the training cluster. Finally, the central server checks if the termination condition is reached based on the evaluation result from clients in this training round. The algorithm pseudo codes are shown in Algorithm 1 and Algorithm 2. 
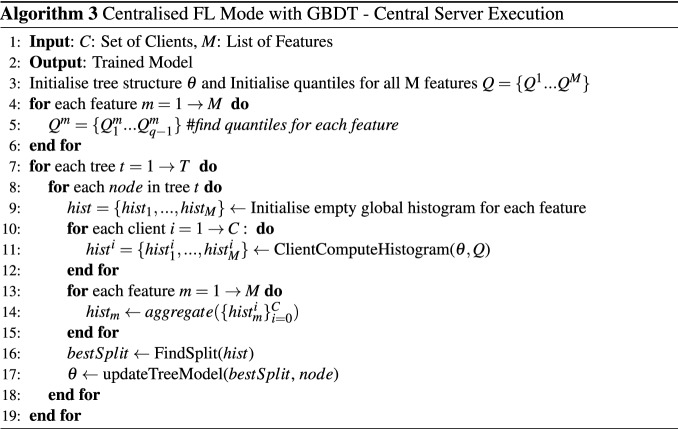

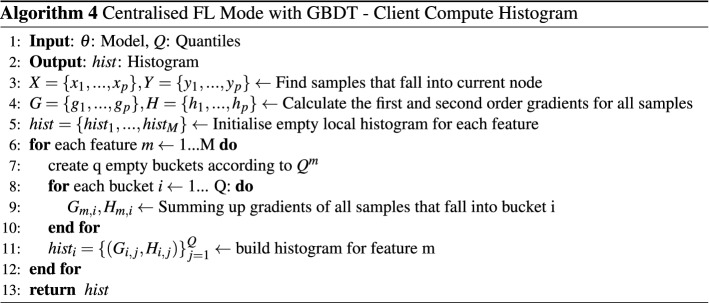


#### GBDT

The critical part of collaboratively constructing a tree model is to find the best split in the feature space point for all clients in the cluster but without sharing their raw data. We implement a federated gradient boosting decision tree model^30^ to achieve this goal, where gradient histograms are shared between clients and the central server and used as training data for model construction. Each such histogram represents the gradient statistics of a specific feature of training data. The histogram is constructed by mapping gradients into multiple buckets. A quantile sketch algorithm^30^ is used to determine $Q-1$ quantile for each feature. Those quantiles are the cut points to divide the range of feature value into *Q* buckets. Similar to the deep neural network model, the GBDT model is built in two parts: Central Server Execution and Client Compute Histogram. The process of training GBDT in centralised federated learning mode is shown in Algorithm 3 and Algorithm 4. At the initialisation phase, the central server defines the training parameters of the tree growth algorithm and coordinates all the clients to run the quantile sketch algorithm to find the quantile of histograms for each feature. Each client computes gradient histograms for each feature during the node split process in parallel by mapping its local training data into buckets according to corresponding feature values of training data. The gradient histograms are transmitted to the central server. Once the central server receives all the gradient histograms, it aggregates each feature’s histograms and searches all the aggregated histograms for the split point. The node is then split into two nodes, and the central server begins to coordinate the splitting of the next node. The tree growth process will be terminated when the stop criteria are met.

### Decentralised federated learning mode

The central server is no longer needed to coordinate the collaborative model construction in the decentralised federated learning mode. Instead, the model is constructed by peer-to-peer communication between clients and the details are shown in Algorithm 5. We assume that clients in a training cluster form a fully connected network, meaning that information can be sent between any two clients. Each client is required to perform both local model training and model aggregation. Before the training begins, each client needs to perform the following steps: initiating the local model parameter using the same random seed, splitting its local dataset into a training set and a test set, and setting up a training protocol for the first round. An asynchronous model aggregation mechanism and dynamic training protocol are proposed to improve the flexibility and security of the framework. The framework allows clients to refine the training protocol on the fly by their network states and available computing resources. The model aggregation can be performed immediately after a client completes its local training process without considering the status of other clients. The requests for the joint model update are randomly sent to *K* other clients in the same cluster during the model aggregation process. The requested clients send out their local models while continuing the training process. After the client has received all models, it uses the local test set to evaluate the performance of all received models and the local models. Each model is allocated with a performance-based weight according to its reaction to the test set. The reciprocal of the error is used as the weight of the model, as the smaller the value of errors in our experiments, the better the model performance. The weight is defined as $\frac{L_{k}^{-1}}{\sum _{i=1}^{K+1}L_{i}^{-1}}$, where $L_k$ is the MAE of the model of client k on the test set of the client currently performing aggregation. The local model is updated by a weighted average of all models, followed by starting a new round of training.
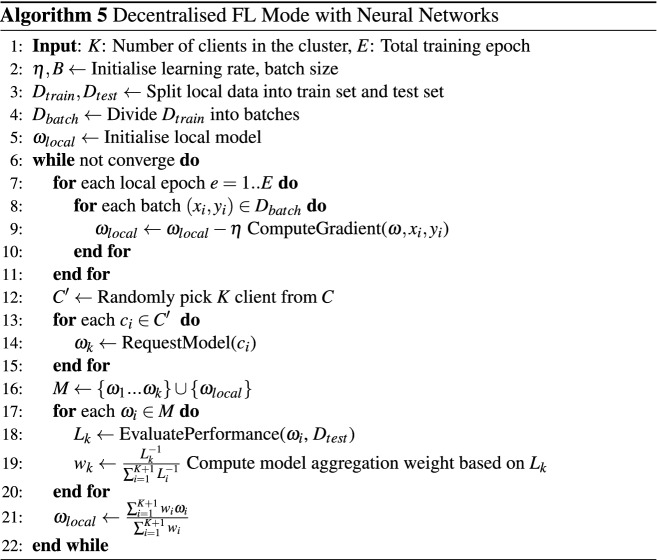


## Results

In this section, we first introduce the dataset, REFIT^31^ (Personalised Retrofit Decision Support Tools For UK Homes Using Smart Home Technology), used for conducting the experiments, followed by the performance metrics used to gauge the quality and utility of our approach. We then present the setup of our experimental studies, including both hardware and software. We conducted comprehensive experiments to evaluate our proposed framework from two perspectives, performance and privacy awareness. For the performance-related evaluations, we carefully examined the training error convergence and NILM disaggregation performance of our proposed federated learning framework in both centralised and decentralised modes. For convenience, we use the term centrally-trained, which refers to those models trained in the centralised mode, and distributively-trained refers to those trained in the decentralised mode. For the privacy awareness evaluations, we studied the effectiveness of a gradient attack on NILM applications in our framework. We demonstrated that the gradient attack is unlikely to acquire valuable information from our framework without explicit privacy protection mechanisms.

### Data

The REFIT electricity load measurement dataset^31^ is one of the four publicly available REFIT datasets. It contains raw electrical consumption data of 20 households in Loughborough, UK, from 2013 to 2015 at both aggregate and appliance levels. The data was measured in watts and sampled at 8-second intervals. We used the datasets from five houses and picked five commonly used appliances, namely, dishwasher, refrigerator, washing machine, microwave oven, and kettle, to form a total of 25 datasets for model training. The sequence-to-point NILM model is built to process the raw electrical consumption data. The aggregated consumption sequences were sliced by a window size of 19 data samples. Each sliced subsequence corresponds to a single appliance level consumption at its middle point. For each of the 25 datasets, 80% of the samples were used for model training and the remainder for testing.

### Experiment setup

We implemented our algorithm with PyTorch on Google Colab, which provides computing resources of an Intel Xeon CPU 4 x 2.30GHz, 16GB RAM, and an NVIDIA Tesla P100 Graphic Card with 16GB VRAM. All experiments were carried out in Ubuntu 18.04. A convolutional neural network (CNN^32^) model with five convolutional layers followed by two linear layers and a gradient boosting decision tree (GBDT^33^) model were used to train sequence-to-point NILM models. The hyper-parameters for training these models are presented in Table 1, unless otherwise stated. All reported data points are an average of 500 executions.

**Table 1:** The parameters for training NILM models.

Parameters for training GBDT model			
Total boosting rounds	100	Maximum tree depth	10
Maximum bins	500	Learning rate	0.25
L1 regularisation	0.02	L2 regularisation	0.0001

Parameters for training CNN model			
Total training rounds	50	Local training epochs	2
Batch size	1024	Optimiser	Adam
Learning rate	0.001	Beta1	0.09
Beta2	0.999	Epsilon	1E-08

### Evaluation metrics

We used the training convergence of the models to evaluate the efficacy and stability of the proposed framework. The training errors are recorded at the end of each training round, and the learning curve is plotted to check the convergence status of different machine learning models. The training loss is evaluated by RMSE, which measures the standard deviation of the training error as defined in Equation (1). RMSE is computationally simple and easily comprehensible to serve as an objective function for model training. We also employed four other performance metrics to evaluate the framework performance from different aspects. The disaggregation performance of NILM models is evaluated by three commonly used metrics, MAE, SAE and NDE, in NILM stuides^34,35^. Mean absolute error (MAE) indicates the average absolute error between model prediction and actual value. It is formally defined as Equation (2) where *y* and $\hat{y}$ represent the predicted value and actual value, respectively. Signal aggregate error (SAE), as shown in Equation (4), measures the relative difference between the total predicted energy consumption and the actual value in any given period *T*. Equation (5) mathematically defines normalised disaggregation error (NDE), which denotes the normalised error between the predicted consumption and the actual readings. Mean relative error (MRE) is used exclusively in privacy leakage evaluation, defined by Equation (3), representing prediction error relative to observed values. It shows the similarity of the recovered data to the actual data to reveal the risk of privacy leakage. For all metrics, the lower the value, the more minor the deviation between estimates and ground truth generated by the model.

1 $$\begin{aligned} RMSE= & {} \sqrt{\frac{1}{N}\sum _{i=1}^{N} y_{i} - \hat{y}_{i}} \end{aligned}$$

2 $$\begin{aligned} MAE= & {} \frac{1}{N}\sum _{i=1}^{N}|y_{i} - \hat{y}_{i}| \end{aligned}$$

3 $$\begin{aligned} MRE= & {} \frac{1}{N}\sum _{i=1}^{N}\frac{|x_i - \hat{x_i}|}{x_i} \end{aligned}$$

4 $$\begin{aligned} SAE= & {} \frac{|\sum _{i=1}^{N}y_{i} - \sum _{i=1}^{N}\hat{y}_{i}|}{\sum _{i=1}^{N}y_{i}} \end{aligned}$$

5 $$\begin{aligned} NDE= & {} \sqrt{\frac{\sum _{i=1}^{N}(y_{i} - \hat{y}_{i})^2}{\sum _{i=1}^{N}y_{i}^2}} \end{aligned}$$

### Centralised federated learning CNN model evaluation

This section evaluates the performance of sequence-to-point NILM models in our proposed framework under the centralised federated learning mode. The experiments were conducted on a training cluster consisting of five clients. The clients are connected via a central server for performing the centralised model training. In each round of training, all clients first update their local models using the private local data, and then the updated models are sent to the central server for aggregation. Please note that the selection of five clients is due to the simplicity of interpreting the results. Each client has a training set of the same size. We also assume that each client is equipped with the same computational resources and follows the same training protocol. The CNN and GBDT models mentioned above were used to perform NILM to identify the operations of the appliances. To benchmark and monitor the performance variation of our framework over time, we also tested the same models trained and running on the local device only to perform the same tasks.

**Figure 2:**
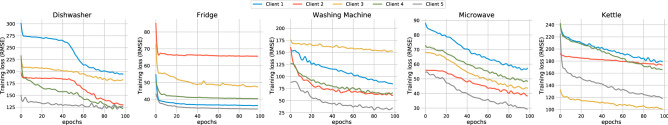
Convergence of training loss for the centrally-trained CNN across five houses on REFIT.

**Figure 3:**
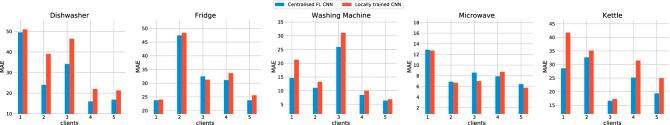
Comparison of MAE between centrally-trained CNN and locally-trained CNN across five houses on REFIT.

**Table 2:** Comparison of disaggregation error on test sets between centrally-trained CNN and locally-trained CNN.

Model	Metrics	Dishwasher	Fridge	Kettle	Microwave	Washing Machine
Locally-trained CNN	MAE	40.2367	33.5251	28.0363	7.8309	15.4629
	SAE	0.0245	0.0194	0.2116	0.2659	0.1776
	NDE	0.8487	0.7012	0.8279	0.9523	0.7608
Centrally-trained CNN	MAE	32.5741	32.5331	24.9767	8.3338	13.7557
	SAE	0.0521	0.0092	0.0281	0.0532	0.0215
	NDE	0.8284	0.6975	0.8567	0.9934	0.7600

Figure 2 shows the training loss convergences of the centrally-trained CNN models in our framework. It can be seen that our framework provides stable training loss convergences on all target appliances. This result suggests that the centrally-trained models have strong generalisation capabilities within the training cluster. The framework can guarantee stable convergence of the loss for the target appliances without compromising any client, regardless of appliance types, the number of appliances and usage patterns. We compared the disaggregation error on test the set between the centrally-trained CNN model and locally-trained CNN in Fig. 3 and Table 2. As shown in Table 2, the centrally-trained CNNs achieve a lower decomposition error on three evaluation metrics than that of locally-trained CNN models for most of the appliances. Figure 3 depicts the MAE of each client on the test set. It can be clearly observed that the MAE of centrally-trained CNN is kept below the locally-trained CNN model in most cases. This result suggests that not only does the centrally-trained CNN achieve an overall lower decomposition error, but all clients in the training cluster can obtain a more accurate energy decomposition model through the centralised federated learning mode. The centrally-trained CNN model actually represents a existing deep learning-based federated learning NILM solution. A similar model structure can be found in^14,36^. It is used as a baseline for the subsequent comparisons.

### Decentralised federated learning CNN model evaluation

In this section, the performance of the NILM models trained in the decentralised federated learning mode is assessed. We conducted the experiments with the same tasks as the centralised federated learning experiments. In the decentralised federated learning mode, each client defines its own training protocol to update the local model asynchronously during the training process. Once a client reaches the model aggregation phase, it acquires models from *k* other clients in the same cluster for model aggregation according to a weighted average of values that reflects the performance of each model on the local test set. In the experiments, we investigated the impact of the choice of *k* on training loss convergence. We then compared the performance of the NILM algorithms trained in centralised federated learning, decentralised federated learning and local modes.

**Figure 4:**
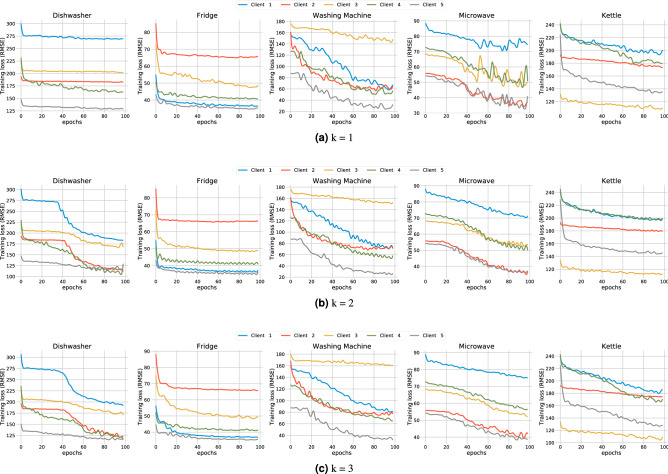
Convergence of training loss for decentralised federated learning mode with different k across five houses on REFIT.

**Figure 5:**
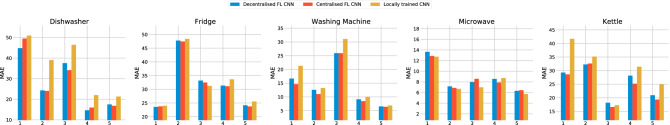
Comparison of MAE among distributively-trained CNN, centrally-trained CNN and locally-trained CNN across five houses on REFIT.

Figure 4 shows the loss convergences of the CNN models trained in the decentralised mode with different *k*. Although the training error of each appliance model is quickly converged in all experiments, a noticeable difference still exists in the local convergence process. Figure 4a depicts the convergence curves when *k* is set to 1. We noticed that rapid fluctuations exist in the convergence curves of each model, which is particularly evident in the washing machine and microwave models. The change of the convergence rate of the models is quite slow, e.g. the dishwasher model was still trapped at a local minimum after 100 rounds of training. However, these issues were mitigated by increasing the value of *k*. Figure 4b,c show the convergence curves when *k* is set to 2 and 3, respectively. We can observe that the curves of the training loss convergence became smoother along with the increase of the *k* value and the model convergence curve showed a tendency to match the curve obtained from the centralised federal learning mode. We also compared the performance of the NILM models trained in the decentralised mode and the centralised mode. We set *k* to be 2 for training the NILM models in the decentralised mode for a fair comparison. Table 3 shows the evaluated performance of the NILM models trained in the decentralised mode on the test sets, and Fig. 5 compares the performance of the NILM models trained in three different modes. We can see that the models trained in the decentralised mode clearly outperform the locally-trained ones and show similar performance to those trained in the centralised mode in terms of accuracy.

**Table 3:** Disaggregation error on test sets for distributively-trained CNN.

Model	Metrics	Dishwasher	Fridge	Kettle	Microwave	Washing machine
Distributively-trained CNN	MAE	27.6742	32.4415	26.7001	8.4139	14.1102
	SAE	0.1525	0.0592	0.1750	0.1255	0.0823
	NDE	0.8400	0.6914	0.8314	0.9999	0.7729

### GBDT model evaluation

**Figure 6:**
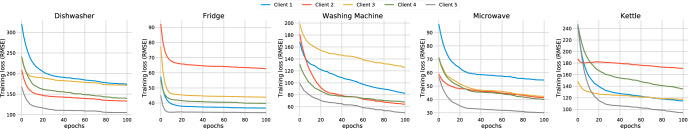
Convergence of training loss for centrally-trained GBDT across five houses on REFIT.

**Figure 7:**
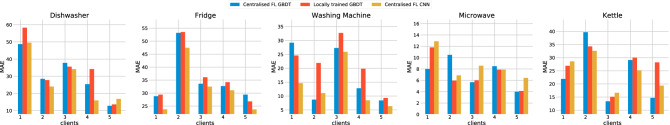
Comparison of MAE between centrally-trained GBDT, locally-trained GBDT, and centrally-trained CNN model across five houses on REFIT.

In this section, we examined the performance of GBDT in centralised training mode for the sequence-to-point NILM problems. We also used locally-trained GBDT models and centrally-trained CNN as benchmarks in the experiments. As shown in Fig. 6, the training loss of the centrally-trained GBDT model on all clients converged quickly (in about 20 epochs or less) to a stable value. Compared to the same test with CNN depicted in Fig. 2, the GBDT models clearly outperformed the CNN ones as the loss curves decrease smoothly and coherently to the stable statuses in noticeably short epochs, This result suggests that the lightweight nature of GBDT requires fewer parameters to fit during training, making the model convergence rapidly. We also compared the performance between the centrally-trained and locally-trained GBDTs. The results are shown in both Fig. 7 and Table 4. Not surprisingly, the results show that the centrally-trained GBDT outperformed the locally-trained one in nearly all aspects. We believe the performance difference comes from the centrally-trained GBDT model can learn extra knowledge from the data of the other members in the training cluster to improve its prediction accuracy. In Fig. 7, we also observed that the GBDT model achieved the state of the art performance. Its performance was equally matched to the CNN model in our tests. More importantly, the GBDT model consumed small computational resources. As shown in Table 5, its model size and inference time are about 1/6 and 1/12 of the CNN model. The above results demonstrate that the GBDT model can provide accurate predictions while requiring significantly fewer computing resources. These unique properties make it the leading candidate for performing NILM on those resource-limited devices.

**Table 4:** Comparison of disaggregation error on test sets between centrally-trained GBDT and locally-trained GBDT.

Model	Metrics	Dishwasher	Fridge	Kettle	Microwave	Washing machine
Locally-trained GBDT	MAE	36.1568	36.8310	27.2049	7.1743	21.7627
	SAE	0.0195	0.0031	0.4791	0.9799	0.1039
	NDE	0.7892	0.7194	0.7878	0.9061	0.8480
Centrally-trained GBDT	MAE	32.1918	36.3702	24.4394	7.1335	19.2950
	SAE	0.0088	0.0063	0.0155	0.0726	0.0639
	NDE	0.7568	0.7278	0.7655	0.8605	0.7804

**Table 5:** Comparison of model size and inference time between centrally-trained GBDT and centrally-trained CNN. Both models were tested 10 times on a test set of 100,000 samples in a single-core CPU setting.

Model	Model size	Inference time
Centrally-trained CNN	4.463MB	11.21s
Centrally-trained GBDT	0.756MB	0.97s

### Training cluster evaluation

In this section, we studied how the clustering algorithm affects the performance of the federated learning model on clients. We used more clients in the cluster experiments to better demonstrate the algorithm. Ten houses were selected from REFIT to represent ten individual energy users and divided into two equal-sized training clusters by the clustering algorithm described before. The CNN model was used to perform NILM tasks in our experiments. We tested the CNN model in three different scenarios, 1) centrally-trained with the data only from the belonging cluster, 2) distributively-trained with the data only from the belonging cluster, and 3) centrally-trained with all data from ten houses. The trained models were tested on the test set of each house. Note that the model trained with data from all ten houses uses twice the training data as the other two models.

**Figure 8:**
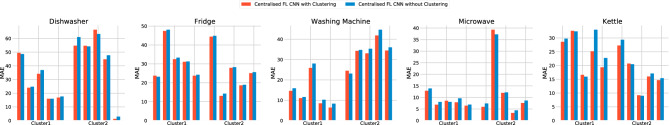
Comparison of MAE between centrally-trained CNN with and without clustering on REFIT.

**Figure 9:**
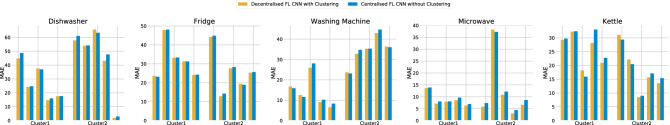
Comparison of MAE between distributively-trained CNN with clustering and centrally-trained CNN without clustering on REFIT.

The experiment results are shown below. Figure 8 shows the MAE comparison between centrally-trained CNN models with and without clustering. It is not hard to see that the prediction error of the model decreased after clustering in most cases. The average MAE of the model trained with clustering dropped from 22.51 to 21.02 compared to the one without clustering. This result indicates that employing a clustering algorithm can help to reduce the discrepancies in the distribution of the grouped user data and improve the overall model performance accordingly. Figure 9 shows the MAE comparison between the distributively-trained CNN model with clustering and the centrally-trained CNN model without clustering. We can again observe a clear performance improvement after clustering. The distributively-trained CNN model trained in each training cluster reduces the average MAE by 0.53 compared to the non-clustered centralised one. Our experiment results indicate that clustering clients with similar statistical distributions can mitigate the impact of non-IID (Independent and Identically Distributed) data on the global model. In addition, we found that the simple increment in clients does not necessarily improve the global model performance. This finding is against the conventional machine learning common sense - the more training data, the better the model performance. However, in federated learning, a simple combination of the clients with non-iid data distribution can slow down the convergence of the global model and sacrifice performance. The naive increase in training data could be counterproductive and will not be the best strategy for performance improvement.

### Privacy leakage evaluation

This section conducted comprehensive experiments to evaluate the effectiveness of gradient attacks on our federated learning framework and analyse the privacy leakage risks for NILM applications.

We start with a brief introduction of the gradient attack, followed by the experimental results.

#### Deep leakage from gradients

Deep Leakage from Gradients (DLG)^37^ is an optimisation-based method that recovers raw training data by continuously adjusting the randomly initialised dummy data and matching its gradient to the observed gradient. The objective function is

$$ {x'}^*,{y'}^* = {{\,\mathrm{arg\,min}\,}}_{x', y'} L(\triangledown W', \triangledown W) = {{\,\mathrm{arg\,min}\,}}_{x', y'} || \frac{\partial \ell (F(x', W), y')}{\partial W} - \triangledown W ||^2$$

where $L(\triangledown W',\triangledown W)$ represents the loss function measuring the similarity between the gradient of dummy data $\triangledown W'$ and the actual gradient $\triangledown W$. $\ell (F(x', W), y')$ is the objective function for deep network training. It only needs to ensure $\ell$ as a differentiable function held for most machine learning tasks. This optimisation problem can then be solved by using a standard gradient-based method.

#### Result

We focus primarily on the centralised training mode, as the decentralised training mode can be somehow seen as each client running a centralised training program. Therefore, the privacy evaluation for the centralised mode is also applicable to the decentralised mode. We used the cosine similarity between the observed and actual gradients as the objective function for the gradient attack. The Adam optimiser was used to solve the optimisation problem. Each experiment ran at least 200,000 iterations to ensure that the loss function converged. We examined the effectiveness of gradient attacks on the recovery of training data under different settings (e.g., batch size, model convergence status, and various machine learning tasks) for federated learning separately. We used the centrally-trained CNN models for the tests as this mode is more vulnerable to privacy leakage. The experiment data were 24 randomly selected datasets equally extracted from the washing machine, fridge, and kettle. We employed MAE, MRE, SAE, and NDE as the performance metrics to measure the quality of the attacks.

**Figure 10:**
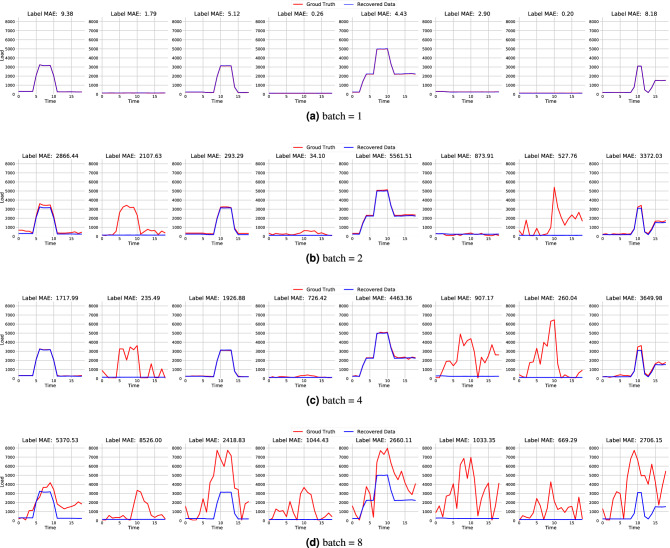
Some of recovered training data from model updates with different batch sizes. The recovered training data were randomly selected from the kettle dataset.

We first investigated the effect of local batch size on a basic scenario where the model is in its initial state without any training. Each client feeds a small batch of data to update the model in the local update phase and then sends the updated model to the central server. Once the central server receives a model from a client, it can derive the gradients of that client in the current training round by calculating the weight differences between the global model and the received one. The central server recovers the client’s raw inputs and labels from the gradient using the DLG algorithm. Table 6 shows the error of the training data recovered from the gradient attack for different tasks under different batch sizes. It is easy to note that the gradient attack can effectively recover the training data when the batch size is small. For example, when the batch size is equivalent to 1 (batch size denoted by B1), the errors of the recovered training inputs and labels are concentrated within a limited range. However, as the batch size increases, the error of the recovered data increases dramatically. When the batch size equals 8, the MAE values between the recovered data and the actual training data reach 994.98 and 986.45 in the classification and regression tests. Meanwhile, the MRE values reach 2.58 and 3.55, respectively. The recovered data errors are even larger than the actual training data values. Under such circumstances, the recovered data can hardly reveal any useful information. To provide a clear demonstration, we show the results of recovered data in Fig. 10. Please note that the gradient does not contain any information about the order of the training data. The recovered data is out of order and cannot be directly compared with the original batch data. As a result, we applied the Hungarian algorithm^38^ to find a match between the recovered and the actual training data so as to evaluate the recovery error on the matching result. It can be seen that when the batch size is 1, the recovered training data matches perfectly with the actual training data. The MAE between the recovered and actual labels is maintained within an acceptable range. As the batch size increases, the MAE gradually increases. When the batch size is 2 and 4, there is a mismatch on the part of the recovered training data, while some of the recovered data still align with the actual training data. When the batch size increases to 8, the gradient attack fails to recover any training data.

**Table 6:** Effectiveness of gradient attack on different NILM tasks with different local batch size.

Experimental		Input recovery				Label Recovery
Task	Batch size	MAE	MRE	SAE	NDE	MAE/Accuracy
Classification	B1	2.5578	0.0103	0.0004	0.0028	100%
Classification	B2	915.6924	1.6454	1.0017	1.6096	100%
Classification	B4	1115.2008	2.8736	1.216	1.8586	87.51%
Classification	B8	994.9842	2.5825	0.9251	1.52	70.83%
Regression	B1	0.0852	0.0003	0.0001	0.0006	0
Regression	B2	351.2712	1.5227	0.3029	0.6359	2679.7218
Regression	B4	653.9442	2.7614	0.7304	1.1266	1679.622
Regression	B8	986.4582	3.5523	1.143	1.3202	3511.8594

We also evaluated whether the effectiveness of the gradient attacks would be affected by the convergence state of the model. The convergence of the model is quantified by the number of epochs in which the model is trained. We set the recovery batch size to 1 and recovered the training data by the models’ weights from 0, 1, 5, and 10 epochs. Table 7 illustrates the recovery performance of gradient attack under different states of model convergence. Both regression and classification tasks are presented. It is not hard to note that the convergence of the model has a significant impact on the gradient attack. When recovering training data from the weights of an untrained model, the discrepancy between the recovered and real data is low. As the training epoch increases from 0 to 10, the MRE value of the recovered input increases from 0.01 to 6.6 for the classification tasks and from 0.0006 to 10.462 for the regression tasks. Also, the accuracy of the recovered training labels decreases significantly. The MAE of the labels grows from 0 to 474.89 for the regression tasks, while the accuracy of the labels drops from 100% to 66.67% for the classification tasks. The results indicate that the gradient attack rapidly loses efficacy in recovering the training data with the epoch increasing. Thus, we can conclude that, for NILM tasks, the gradient attack method only works in the very early stages of NILM model training, but such leakage is shallow and not sufficient to pose a threat to user privacy.

**Table 7:** Effectiveness of Gradient Attack on different NILM tasks with different model convergence state size.

Experimental		Input recovery				Label recovery
Task	Epoch	MAE	MRE	SAE	NDE	MAE/accuracy
Classification	0	2.5578	0.0105	0.0001	0.003	100%
Classification	1	899.493	4.6346	0.8739	1.5489	91.67%
Classification	5	765.6348	3.4149	0.5519	1.1988	79.17%
Classification	10	1268.6688	6.6326	1.3876	1.7306	66.67%
Regression	0	0.0921	0.0006	0	0.0008	0
Regression	1	882.441	3.5588	0.8478	1.3624	446.7624
Regression	5	1081.0968	4.1875	0.9005	1.3197	509.8548
Regression	10	2302.8726	10.4625	2.8196	2.6742	474.8982

## Discussion

In this work, we propose a model-agnostic hybrid federated learning framework for NILM applications in sustainable smart cities. It aims to provide a flexible, efficient, and secure way to train NILM models collaboratively. The core idea of the framework is to let every user use the best-suited NILM models introduced in the appropriate environment to meet their needs. Both centralised and decentralised federated learning is supported in our framework. In the centralised federated learning mode, the server in each training cluster is responsible for provisioning and managing the training process for all users. This training mode has many advantages, such as fast convergence of the global model, good generalisation, and low communication costs. Besides, the energy wholesalers and retailers can utilise real-time information from their fellow users to better understand their behaviours and activities. They can align with the dominant understanding of users as rational individuals to set up more attractive financial incentives for participating in demand response^39^ programs, which have been acknowledged as a viable solution to ensure grid stability and security of power supply. Despite the versatility of the centralised federated learning mode, it encounters multiple issues at the system level, such as single point failure and poor scalability. In addition, the server could quickly turn into a performance bottleneck of the framework. As the number of users increases, the communication and computation load on the server increases rapidly. The time required for training per round also increases. In the decentralised federated learning mode, the users in the same training cluster share the models asynchronously with others via peer-to-peer communication, and each user is only responsible for their models. This mode improves the scalability and elasticity of the framework. Our framework currently supports the training of neural network models in both centralised and decentralised modes and gradient boosting tree models in centralised mode. We tested the performance of two machine learning models using our proposed framework on a real-world dataset and compared it with locally trained models. The experimental results show that the models trained in our framework outperform the locally trained models in terms of accuracy and diversity. Also, the models trained in the decentralised mode have similar convergence speed and performance to those trained in the centralised mode.

We have also investigated the user privacy issues in federated learning for NILM applications. As mentioned previously, the leakage of an electrical consumption dataset can reveal behavioural patterns of energy users and seriously compromise their privacy. Therefore, we investigate the effectiveness of a state-of-the-art attack method against federated learning frameworks in NILM applications. Through our experiments, we came up with two findings. The first is that gradient attack is only applicable to centralised federal learning frameworks. To perform the gradient attack, the attacker must know updated gradients and the size of the local dataset used for training. Such information is only available to a central server in the centralised federated learning mode. In a decentralised federated learning mode, the central server is no longer used, and asynchronous model updates are employed. An attacker masquerading as a client has access to models from only a few random clients, and they have no way of knowing the size of the local dataset used for each model update. Therefore, gradient attack can hardly be applied to a decentralised federated learning framework. Although a gradient attack can be used to attack a centralised federated learning framework, this does not mean that it can compromise user privacy. We show that gradient attack is only valid to recover some fragments of electrical consumption data used for training under certain conditions, such as in the early stages of model training and when a very small training batch size is chosen. These limitations make almost impossible for gradient attack to compromise any user privacy in practice. We have good reason to believe that the gradient attack is not effective in violating user privacy in our proposed framework. Furthermore, we consider it unnecessary to use encryption or add noise to prevent gradient attack in federated learning for NILM applications. However, previous studies have come to the opposite conclusion. They experimentally show that gradient attack has a satisfactory recovery accuracy in image processing tasks and suggest that precautions need to be taken to prevent gradient attack. This makes us wonder why gradient attack do not work well on NILM tasks. We believe that there are two reasons behind the contradiction. First, image data usually describes real-world objects, which are more easily understood by people. So even if the accuracy of the reconstructed data is not as high, one can still guess what is in the image by associating the partially recovered image fragments with known real-world objects. Secondly, the specificity of image recognition tasks, for example in face recognition tasks where each participant holds a person’s face data, gives gradient attack more opportunities to steal the user’s facial features from the batch training data. These reasons make gradient attack a higher risk of privacy violation on image datasets.

This work presents our preliminary results in realising a model-agnostic hybrid federated learning framework for NILM applications. In the future, we aim to implement an end-to-end federated learning framework comprising a complete training process from data pre-processing, model training and deployment. We will integrate more machine learning models and more federated learning modes into our framework to handle various smart city applications. We will also optimise our decentralised federated learning framework by improving the convergence speed of models and the overall communication efficiency.

## Data Availability

The datasets analysed during the current study are available in the REFIT repository https://www.refitsmarthomes.org/datasets/.
